# miR-129 Regulates Yak Intramuscular Preadipocyte Proliferation and Differentiation through the PI3K/AKT Pathway

**DOI:** 10.3390/ijms25010632

**Published:** 2024-01-03

**Authors:** Chunyu Qin, Hui Wang, Jincheng Zhong, Hongbiao Ran, Wei Peng

**Affiliations:** 1 Qinghai Academy of Animal Science and Veterinary Medicine, Qinghai University, Xining 810016, China; 220710002005@stu.swun.edu.cn; 2 Key Laboratory of Qinghai-Tibetan Plateau Animal Genetic Resource Reservation and Utilization, Sichuan Province and Ministry of Education, Southwest Minzu University, Chengdu 610225, China; wanghui892321@swun.edu.cn (H.W.); 22100047@swun.edu.cn (J.Z.); 200710002007@stu.swun.edu.cn (H.R.)

**Keywords:** miR-129, PI3K/AKT, yak, adipocytes, differentiation, proliferation

## Abstract

miR-129 plays a crucial role in regulating various cellular processes, including adipogenesis; however, its downstream molecular mechanisms remain unclear. In this study, we demonstrated that miR-129 promotes yak adipogenesis in vitro via the PI3K/AKT pathway. Overexpression and interference of miR-129 in yak intramuscular preadipocytes (YIMAs) enhanced and inhibited cell differentiation, respectively, with corresponding changes in cell proliferation. Further investigation revealed that miR-129 enhances AKT and p-AKT activity in the AKT pathway without affecting cell apoptosis, and a specific inhibitor (LY294002) was used to confirm that miR-129 regulates YIMAs proliferation and differentiation through the PI3K/AKT pathway. Our findings suggest that miR-129 promotes yak adipogenesis by enhancing PI3K/AKT pathway activity. This study provides the foundation to precisely elucidate the molecular mechanism of miR-129 in YIMAs adipogenesis and develop advanced miRNA-based strategies to improve meat nutrition and obesity-related ailments in beef production.

## 1. Introduction

Yaks (*Bos grunniens*), a unique breed of livestock in the Qinghai–Tibetan Plateau region, hold a significant position in China’s beef market. Yaks are the third largest breed of cattle in China, accounting for over 95% of the world’s yak population [1]. Studies indicate that fat content, particularly intramuscular fat (IMF) content, plays a significant role in the tenderness, juiciness, and flavor of meat [2,3]. However, the low IMF content of yak meat has become a major bottleneck, restricting high-quality meat development in the yak industry. Recent studies report that non-coding RNAs (ncRNAs) regulate key aspects of lipid metabolism [4,5,6,7].

MicroRNAs (miRNAs) are a subclass of ncRNAs consisting of approximately 20 nucleotides that play crucial roles in regulating adipocyte proliferation, differentiation, and apoptosis [8]. In particular, miR-129-5p has been identified as a potential biomarker because it is a crucial tumor suppressor [9,10]. Several studies have highlighted the significant regulatory potential of miR-129 in lipid metabolism and adipogenesis and its potential as a biomarker and therapeutic target for obesity [11,12,13,14]. In our previous studies on adipogenesis in YIMAs, we noted that miR-129 was significantly down-regulated after overexpression of the adipogenesis-promoting factor lncFAM200B [4]. This suggests that miR-129 may have a key regulatory role in yak adipogenesis, but further experimental validation is required.

The PI3K/AKT pathway is crucial for insulin stress, metabolic regulation, and cell apoptosis [15]. Several studies have demonstrated that miR-129 regulates cell growth and development by participating in the PI3K/AKT pathway, either at the post-transcriptional or post-translational level. For example, miR-129-5p overexpression inhibits the development of retinoblastoma by directly targeting *PAX6* via the PI3K/AKT signaling pathway [16]. Similarly, the miR-129-5p/*SLC2A3* axis regulates glucose metabolism and gastric cancer growth and is influenced by the PI3K/AKT pathway [17]. Additionally, Xu et al. showed that miR-129 inhibits prostate cancer cell proliferation and metastasis by targeting *ETS1* via the PI3K/AKT/mTOR pathway [18]. Therefore, we investigated the potential interactions between miR-129 and the PI3K/AKT pathway during the proliferation and differentiation of YIMAs.

In this study, we performed overexpression and interference techniques to manipulate miR-129 levels and investigated its regulatory effects on lipid deposition and the PI3K/AKT signaling pathway in yaks to further explore the relationship between miR-129 and the activity of the PI3K/AKT pathway during YIMAs proliferation and differentiation. The current work provides valuable insights and a theoretical basis for understanding the molecular mechanism of miR-129 in adipogenesis and exploring its biological functions.

## 2. Results

### 2.1. miR-129 Promotes Differentiation of YIMAs

**Figure 1:**
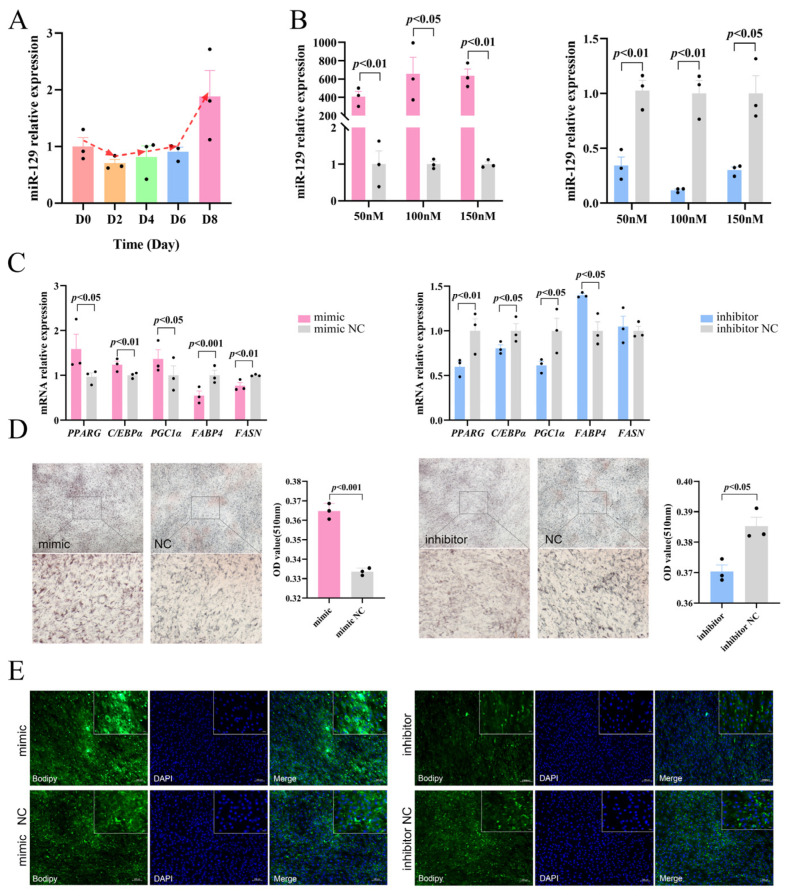
Effects of miR-129 on YIMAs differentiation and lipid deposition. (**A**) Expression of miR-129 in different stages of YIMAs differentiation. (**B**) Concentration screening of miR-129 mimic and inhibitor after miR-129 overexpression and interference. (**C**) mRNA expression of differentiation genes. (**D**) Oil Red O staining. Scale bar: 200 μm, 50 μm. (**E**) Bodipy staining. Scale bar: 100 μm, 20 μm.

To preliminarily investigate the regulatory role of miR-129 in YIMAs adipogenesis, we measured the miR-129 expression levels during YIMAs differentiation. The results showed that the expression of miR-129 gradually increased, indicating that miR-129 has a potential regulatory role in YIMA differentiation (Figure 1A). Subsequently, we manipulated the miR-129 expression in YIMAs using transfection mimics and inhibitors. An optimal concentration of 100 nM was selected for subsequent tests. (Figure 1B). We further analyzed the effects of miR-129 overexpression and knockdown on YIMAs differentiation and lipid deposition in vitro. The reverse transcriptase–-quantitative polymerase chain reaction (RT-qPCR) results showed that overexpression of miR-129 increased the expression of differentiation marker genes (*PPARG* and *C/EBPα*), while interference with miR-129 decreased the mRNA expression of these marker genes. *PGC1α*, a coactivator of *PPARG*, showed a similar trend (Figure 1C). Additionally, we observed that the overexpression of miR-129 suppressed the expression of *FABP4* and *FASN*, which are key targets of *PPARG* during fatty acid synthesis. In contrast, miR-129 interference upregulated the mRNA expression of *FABP4*. Furthermore, Oil Red O and Bodipy staining experiments revealed that overexpression of miR-129 significantly enhanced lipid deposition in YIMAs during differentiation, whereas interference with miR-129 inhibited lipid deposition (Figure 1D,E). These results suggest that miR-129 plays a positive role by regulating the expression of lipid-related genes during YIMAs lipid deposition.

### 2.2. miR-129 Inhibits Proliferation of YIMAs

**Figure 2:**
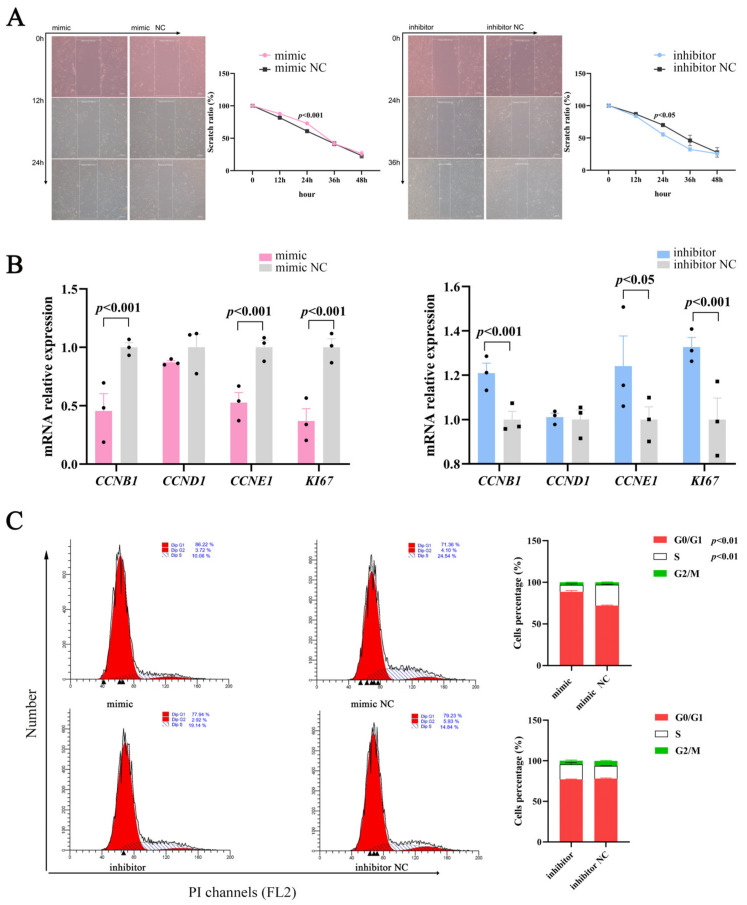
Effect of miR-129 on YIMAs proliferation after miR-129 overexpression and interference. (**A**) Scratch test. Scale bar: 200 μm. (**B**) mRNA expression of proliferative genes. (**C**) Flow cytometry. ▲: The peak with the highest DNA content in G1 or G2.

Cell proliferation and differentiation are highly coordinated processes [19]; therefore, we studied the effects of miR-129 on proliferation during YIMAs differentiation. The scratch results showed that proliferation was significantly altered in the overexpression or inhibition groups compared to their respective controls (Figure 2A). Moreover, miR-129 overexpression significantly suppressed the expression of cell-proliferation-associated marker genes, whereas miR-129 inhibition had the opposite effect (Figure 2B). Overexpression of miR-129 resulted in cell cycle arrest in the G0/G1 phase and decreased the number of cells in the S phase. Conversely, interference with miR-129 had the opposite effect on the number of cells in the G0/G1 and S phases (Figure 2C). These results indicated that miR-129 may impede YIMAs proliferation by inhibiting the transition of cells from the G0/G1 phase to the S phase.

### 2.3. miR-129 Positively Regulates PI3K/AKT Pathway Activity in YIMAs

**Figure 3:**
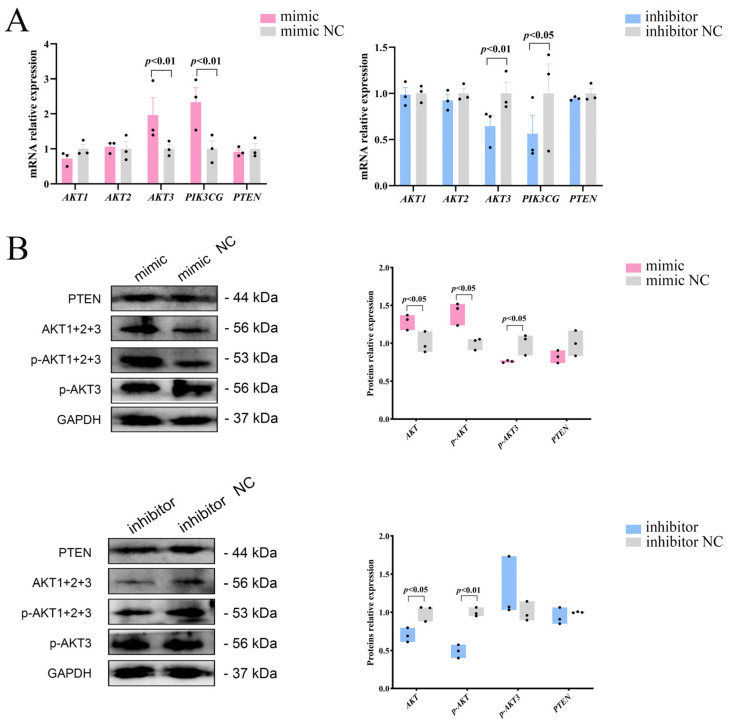
Effects of miR-129 on PI3K/AKT pathway after miR-129 overexpression and interference. (**A**) mRNA expression of *AKT1*, *AKT2*, *AKT3*, *PTEN*, and *PIK3CG*. (**B**) Protein expression of AKT, p-AKT, p-AKT3, and PTEN.

To investigate whether miR-129 influences the proliferation and differentiation of YIMAs via the PI3K/AKT pathway, we initially analyzed the mRNA expression of three isoforms of *AKT* (*AKT1*, *AKT2*, and *AKT3*) after overexpression or interference with miR-129. We observed significant upregulation and downregulation of *AKT3* mRNA expression upon overexpression and interference, respectively. As a nonclassical activator of the PI3K/AKT pathway, *PIK3CG* showed the same expression pattern as AKT3 (Figure 3A). Western blot results showed that miR-129 overexpression increased the expression of AKT and p-AKT proteins but decreased the expression of p-AKT3 protein. In contrast, miR-129 inhibition significantly reduced the expression of AKT and p-AKT proteins, with no significant change in the expression of p-AKT3 (Figure 3B). This suggests that miR-129 has different effects on the mRNA and protein levels of AKT isoforms in the PI3K/AKT pathway.

### 2.4. miR-129 Has No Significant Effect on Apoptosis of YIMAs

**Figure 4:**
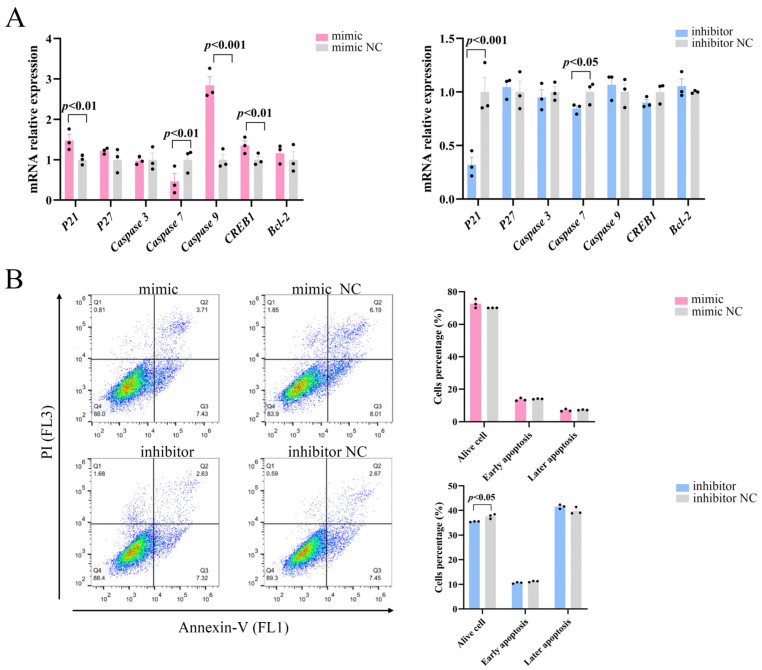
Effect of miR-129 on YIMAs apoptosis after miR-129 overexpression and interference. (**A**) mRNA expression of apoptotic regulatory factors. (**B**) Flow cytometry.

The role of the PI3K/AKT pathway in controlling apoptosis and survival is well-established [20]. We examined whether miR-129 also affects apoptosis in YIMAs via the PI3K/AKT pathway and regulates cell proliferation by examining the expression of essential apoptosis regulators downstream of PI3K/AKT. The results demonstrated that overexpression of miR-129 significantly increased the mRNA expression levels of the apoptosis regulators *P21*, *Caspase9*, and *CREB1* while reducing the expression of *Caspase7*. Simultaneously, inhibition of miR-129 led to a decrease in the *P21* and *Caspase7* mRNA expression levels (Figure 4A). Flow cytometric analysis showed that miR-129 overexpression did not significantly affect early or late apoptosis. However, miR-129 interference resulted in a reduction in the number of viable cells but did not have a significant impact on the ratio of apoptotic cells (Figure 4B). This difference could be attributed to the interference of cell debris during the experiment. Overall, these findings suggested that miR-129 does not significantly impact apoptosis.

### 2.5. miR-129 Promotes Differentiation and Inhibits the Proliferation of YIMAs through the PI3K/AKT Pathway

**Figure 5:**
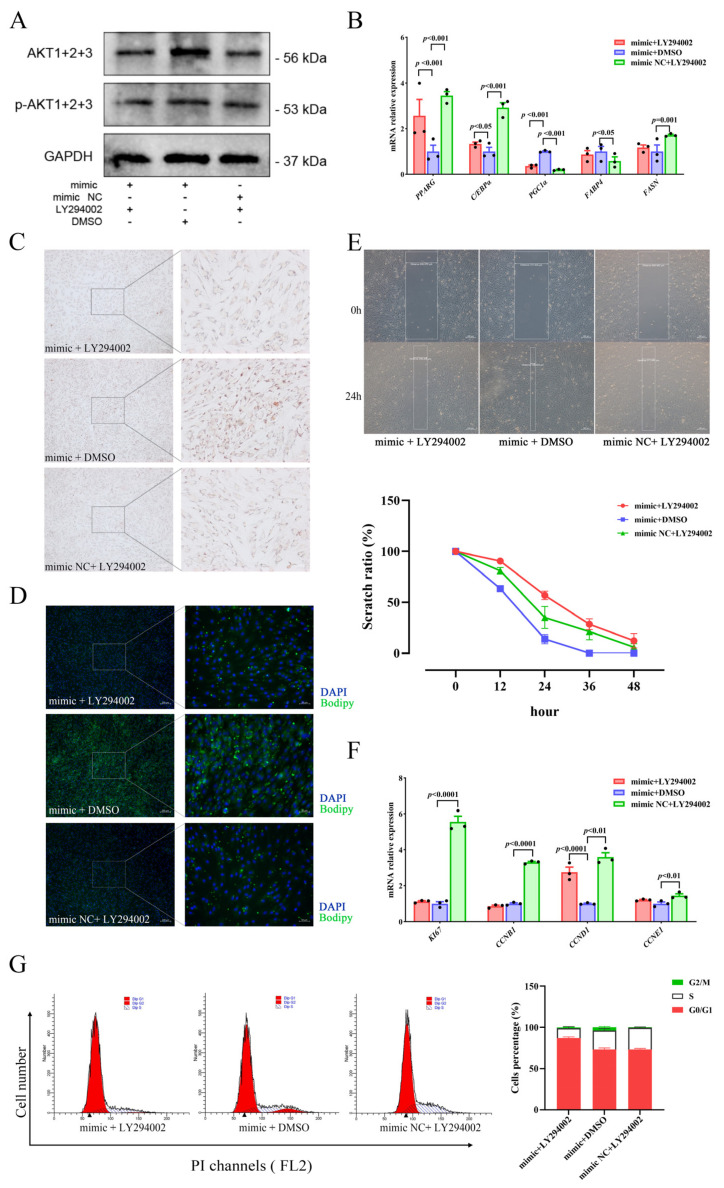
miR-129 regulates YIMAs proliferation and differentiation through the PI3K/AKT pathway after co-transfection of miR-129 mimic or NC and LY294002 or DMSO. (**A**) Protein expression of AKT and p-AKT. (**B**) mRNA expression of differentiation genes. (**C**) Oil Red O staining. Scale bar: 200 μm, 50 μm. (**D**) Bodipy staining. Scale bar: 200 μm, 50 μm. (**E**) Scratch assay. Scale bar: 200 μm. (**F**) mRNA expression of proliferation genes. (**G**) Flow cytometry. ▲: The peak with the highest DNA content in G1 or G2.

To investigate the relationship regarding regulation between miR-129 and PI3K/AKT pathway activity in YIMAs proliferation and differentiation, we inhibited the PI3K/AKT pathway activity after overexpressing miR-129. As expected, the results showed that overexpression of miR-129, followed by injection of LY294002 (a PI3K/AKT pathway inhibitor), significantly suppressed the expression of AKT and p-AKT proteins (Figure 5A). Meanwhile, RT-qPCR results revealed that inhibiting the PI3K/AKT pathway after miR-129 overexpression significantly upregulated *PGC1α* expression (Figure 5B). Additionally, Oil Red O and Bodipy staining results indicated that inhibiting the PI3K/AKT pathway after miR-129 overexpression significantly impeded the enhancing effect of miR-129 on cell differentiation (Figure 5C,D). Scratch assay and flow cytometry results indicated an intensification of the inhibitory impact of miR-129 on cell proliferation (Figure 5E–G). Overall, our findings suggest that miR-129 regulates cell differentiation and proliferation by modulating the activity of the PI3K/AKT pathway, and *PGC1α* may be involved.

## 3. Discussion

Lipid deposition, particularly in intramuscular fat, is a crucial factor influencing beef quality and is considered one of the most economically significant traits in beef production [21]. This process is primarily controlled by preadipocyte turnover, which involves cell proliferation, differentiation, and apoptosis [22]. NcRNAs, particularly miRNAs, have been found to play crucial roles in regulating adipocyte proliferation, differentiation, and apoptosis [23,24,25,26]. miR-129 has now been identified as a potential biomarker of tumor cell growth and development. Numerous studies have demonstrated that miR-129 indirectly regulates adipose tissue development and metabolism [27,28,29,30,31]. In our previous study, we observed that the expression of miR-129 significantly decreased after the overexpression of lncFAM200B, which promotes adipogenesis, and that the expression of miR-129 exhibited an upward trend during the differentiation of YIMAs. These findings suggest that miR-129 plays a role in intramuscular lipid deposition in yaks. We conducted miR-129 overexpression and knockdown experiments using YIMAs. Our results revealed that miR-129 promoted the differentiation and inhibited the proliferation of YIMAs, as observed through Oil Red O, Bodipy, and flow cytometry experiments. However, miR-129 did not affect apoptosis.

The PI3K/AKT signaling pathway, known for its role in insulin signaling, is closely associated with adipogenesis [32]. Although previous studies on yaks have primarily focused on fibroblast proliferation [33], intestinal inflammation [34], and reproduction [35], there is limited research on fat metabolism. Numerous studies have indicated that miR-129 is involved in regulating the PI3K/AKT pathway [16,17,18]. Based on these findings, we proposed that miR-129 affects lipid deposition in yaks via the PI3K/AKT signaling pathway. As expected, our experiments showed that miR-129 positively regulates the activity of the PI3K/AKT pathway by promoting AKT phosphorylation, thereby promoting YIMAs cell differentiation and inhibiting proliferation. miRNAs function by inhibiting translation or causing the degradation of messenger RNA (mRNA) [36]. The *PTEN*/PI3K/AKT pathway plays a crucial role in cell proliferation, apoptosis, and tumor growth [37]. In a study involving rats with chronic heart failure, miR-129-5p was found to inhibit PTEN ubiquitination and enhance PTEN expression by targeting Smurf1 [38]. The restoration of cardiac function led us to investigate whether miR-129 regulates yak lipid deposition by influencing the PI3K/AKT pathway via PTEN. However, we discovered that the impact of miR-129 on intramuscular fat lipid deposition was not influenced by PTEN (Figure 3A,B) and that this inconsistency may be species-specific.

To investigate the relationship between miR-129 and the PI3K/AKT pathway in regulating the proliferation and differentiation of YIMAs, we identified 290 putative target genes of miR-129 by analyzing the overlapping intersections of three databases (miRDB, TargetScan, and miRWalk). However, we did not find any typical targets related to the PI3K/AKT pathway, indicating that miR-129 regulates this pathway indirectly. Instead, we identified the classical pathways that regulate adipocyte differentiation and lipid metabolism. These pathways included the Wnt, calcium, Hippo, and cAMP signaling pathways (Table S1). Notably, the cAMP signaling pathway is considered to be upstream of the PI3K/AKT pathway. We observed that *CAMK4*, a serine/threonine kinase family member, is enriched in the cAMP signaling pathway and regulates the expression of various genes [39,40]. Increased lipid deposition in tissues is closely associated with insulin resistance, and *CAMK4* enhances insulin sensitivity and indirectly modulates lipid metabolism [41,42,43,44]. Furthermore, studies have shown that miR-129 can target the *CAMK4* and that PI3K/AKT acts as a downstream mediator of *CAMK4* in saffronin signaling [45,46]. Therefore, we hypothesized that miR-129 affects lipid deposition through the cAMP-PI3K/AKT signaling pathway involving *CAMK4*. However, the exact mechanism underlying this process requires further experimental validation.

In this study, we observed that miR-129 affects *PPARG*, its coactivators (*PGC1α*), and downstream target genes (*FABP4*), indicating that miR-129 may regulate the expression of downstream target genes through *PPARG* and subsequently regulate adipose deposition in yaks. However, inhibition of the PI3K/AKT pathway led to increased *PPARG* expression when miR-129 was overexpressed, suggesting that miR-129 independently regulates intramuscular lipid deposition in yaks through the PI3K/AKT pathway without involving *PPARG*. The upregulation of *PPARG* may result from the disrupted lipid balance caused by the inhibition of the PI3K/AKT pathway. It is worth noting that *PGC1α*, a downstream target of the PI3K/AKT signaling pathway, is positively influenced by the phosphorylated PI3K/AKT signaling pathway, which enhances its activity [47]. Conversely, when the PI3K/AKT pathway was inhibited, the expression of *PGC1α* decreased; this conclusion is consistent with the results of our analysis. Based on these findings, we propose that miR-129 may regulate lipid deposition in yaks by influencing the PI3K/AKT pathway and subsequently modulating the expression of *PGC1α*. However, further investigations are required to elucidate this mechanism fully.

In conclusion, our study showed that miR-129 promotes the differentiation of YIMAs and inhibits their proliferation by activating the PI3K/AKT pathway. This study provides a theoretical foundation for further exploration of the regulatory mechanisms of miRNAs in the adipogenesis of YIMAs.

## 4. Materials and Methods

### 4.1. Cell Culture and Transfection

Based on a previous study [48], yak preadipocytes were isolated from the *longissimus dorsi* muscle tissue. The cells were then cultured in a complete culture medium consisting of Dulbecco’s modified Eagle medium (DMEM/F12) (HyClone, Logan, UT, USA), 10% fetal bovine serum (Gibco, Waltham, MA, USA), and 1% penicillin–streptomycin (Boster, Wuhan, China) under sterile conditions at 37 °C and 5% CO_2_ until they reached a cell density of 80–90%. Transfection was performed using the Lipofectamine 3000 reagent (Invitrogen, Carlsbad, CA, USA) following the manufacturer’s instructions. The miR-129 mimic, inhibitor, and corresponding NC were synthesized by Tsingke Biotech (Tsingke, Beijing, China). The miR-129 sequence was available in the NCBI for Biotechnology Information BioProject database (accession number PRJNA831897). The treated YIMAs were incubated in either a complete culture medium or induced differentiation medium (complete culture medium containing 100 µM oleic acid) (Sigma Aldrich, St. Louis, MO, USA) for 48 h, after which mRNA and protein expression were detected. The primer sequences are provided in Table S2.

### 4.2. RT-qPCR

Total RNA was extracted using TRIzol (Takara, Shiga, Japan), and complementary DNA (cDNA) was prepared using the PrimeScript RT Reagent Kit (Takara). Stem-loop reverse transcription (RT) primers were designed based on previous studies on miRNA and U6 cDNA synthesis [49]. RT-qPCR was conducted using the SYBR Premix Ex Taq kit (Takara) following the manufacturer’s instructions. Gene expression levels and miRNA levels were normalized using the 2^−∆∆Ct^ method with *GAPDH* and *U6* as internal references, respectively. The primer sequences are shown in Table S3.

### 4.3. Oil Red O and Bodipy Stain

In 6-well plates, cells were seeded and transfected with the miR-129 mimic or inhibitor (with their respective controls). Additionally, the miR-129 mimic was co-transfected with either the PI3K/AKT pathway inhibitor, LY294002 (Beyotime, Shanghai, China), or dimethyl sulfoxide (Boster) to investigate the relationship between miR-129 and the PI3K/AKT pathway in regulating the proliferative differentiation of YIMAs. Differentiated and mature YIMAs were washed thrice with PBS and fixed with 4% paraformaldehyde (Biosharp, Hefei, China) at room temperature for 1 h. After removing the fixative with PBS, the cells were stained with either Oil Red O (Sigma Aldrich) or Bodipy stain (Invitrogen) at room temperature for 30 min each and micrographed (Carl Zeiss, Oberkochen, Germany). Next, 200 µL of isopropanol was added to each well to extract the Oil Red O staining solution, and the optical density (OD) value was measured at 510 nm.

### 4.4. Scratch Test

A scratch assay was used to examine the impact of each treatment on cell proliferation, and the transfection pattern was consistent with the Oil Red O and BODIPY staining. Uniform scratching along the central axis of the well plate was performed using a sterile pipette tip after transfection. The scratch width was measured every 12 h to calculate the scratch ratio.

### 4.5. Flow Cytometry

Flow cytometry was conducted using Sysmex Cube 8.0 (Sysmex, Kobe, Japan). Transfection was performed in accordance with Oil Red O and Bodipy staining. For apoptosis analysis, cells were collected after digestion with 0.25% trypsin (Boster), washed with pre-cooled PBS, centrifuged at 1500 rpm for 5 min, and stained with the Annexin V-FITC/PI Apoptosis Detection Kit (Vazyme, Nanjing, China) at room temperature. For cell cycle analysis, the washed cells were fixed with 70% anhydrous ethanol at 4 °C overnight. After washing with pre-cooled PBS solution, 50 ng/mL PI reagent (Solarbio, Beijing, China) was added and incubated at room temperature with light protection for 30 min before assay. Flow cytometry data were de-adhered and graphically fitted using ModFit 32 software, whereas flow apoptosis data were analyzed using FlowJo (v10) software.

### 4.6. Western Blot

Total cellular proteins were extracted using cell lysis buffer containing protease inhibitors (Boster) and phosphatase inhibitors (Biosharp) for western blotting and IP (Beyotime). The protein content of each sample group was determined using the BCA protein quantification kit (Thermo Fisher Scientific, Waltham, MA, USA). Subsequently, 20 μg of protein from each group of samples was separated on 8% sodium dodecyl sulfate–polyacrylamide gel electrophoresis (SDS-PAGE) and transferred to a polyvinylidene fluoride (PVDF) membrane. Then, 5% skim milk powder was used to seal at room temperature for 1.5 h. After sealing, the PVDF membrane was treated with primary antibodies (Anti-phospho-PIK3CG (Bioss, Beijing, China, 1:2000), Anti-PTEN (Bioss, 1:2000), Anti-AKT1+2+3 (Bioss, 1:2000), Anti-phospho-AKT1+2+3 (Bioss, 1:2000), Anti-phospho-AKT3 (Bioss, 1:2000), GAPDH Antibody (Affinity, Liyang, China, 1:2000), and secondary antibodies (Goat Anti-Rabbit IgG H&L (HRP), Abcam, Cambridge, England, 1:5000). Photographic observations were made using an ultrasensitive ECL chemiluminescent substrate (Biosharp). Western blot results were analyzed using ImageJ v1.54d software (National Institute of Health, Bethesda, MD, USA).

### 4.7. Statistical Analysis

Three independent biological samples were used for each experiment. Student’s *t*-test or one-way analysis of variance (ANOVA) was performed using SPSS version 25.0 (IBM Corp, Armonk, NY, USA). Duncan’s test was employed to determine significance, and values are presented as mean ± standard error of the mean (SEM). The data were visualized using GraphPad Prism v8.0 (GraphPad Software, La Jolla, CA, USA).

## Data Availability

All data generated or analyzed during this study are included in this published article. The data that support the findings of this study are available from the corresponding author upon request.

## Supplementary Materials

The following supporting information can be downloaded at: https://www.mdpi.com/article/10.3390/ijms25010632/s1.
